# On the genome base composition of teleosts: the effect of environment and lifestyle

**DOI:** 10.1186/s12864-016-2537-1

**Published:** 2016-03-02

**Authors:** Andrea Tarallo, Claudia Angelini, Remo Sanges, Mitsuharu Yagi, Claudio Agnisola, Giuseppe D’Onofrio

**Affiliations:** Genome Evolution and Organization – Department BEOM, Stazione Zoologica Anton Dohrn, Villa Comunale, 80121 Naples, Italy; Istituto per le Applicazioni del Calcolo “Mauro Picone” – CNR, Via Pietro Castellino, 111, 80131 Naples, Italy; Faculty of Fisheries, Nagasaki University, 1-14 Bunkyo, Nagasaki, 852-8521 Japan; Department of Biology, Complesso Universitario di Monte Sant’Angelo, University of Naples Federico II, Edificio 7, Via Cinthia, 80126 Naples, Italy

**Keywords:** GC content, Routine metabolic rate, Gill area, Migration, Freshwater, Seawater, Gene expression

## Abstract

**Background:**

The DNA base composition is well known to be highly variable among organisms. Bio-physic studies on the effect of the GC increments on the DNA structure have shown that GC-richer DNA sequences are more bendable. The result was the keystone of the hypothesis proposing the metabolic rate as the major force driving the GC content variability, since an increased resistance to the torsion stress is mainly required during the transcription process to avoid DNA breakage. Hence, the aim of the present work is to test if both salinity and migration, suggested to affect the metabolic rate of teleostean fishes, affect the average genomic GC content as well. Moreover, since the gill surface has been reported to be a major morphological expression of metabolic rate, this parameter was also analyzed in the light of the above hypothesis.

**Results:**

Teleosts living in different environments (freshwater and seawater) and with different lifestyles (migratory and non-migratory) were analyzed studying three variables: routine metabolic rate, gill area and genomic GC-content, none of them showing a phylogenetic signal among fish species. Routine metabolic rate, specific gill area and average genomic GC were higher in seawater than freshwater species. The same trend was observed comparing migratory versus non-migratory species. Crossing salinity and lifestyle, the active migratory species living in seawater show coincidentally the highest routine metabolic rate, the highest specific gill area and the highest average genomic GC content.

**Conclusions:**

The results clearly highlight that environmental factors (salinity) and lifestyle (migration) affect not only the physiology (i.e. the routine metabolic rate), and the morphology (i.e. gill area) of teleosts, but also basic genome feature (i.e. the GC content), thus opening to an interesting liaison among the three variables in the light of the metabolic rate hypothesis.

**Electronic supplementary material:**

The online version of this article (doi:10.1186/s12864-016-2537-1) contains supplementary material, which is available to authorized users.

## Background

The DNA base composition is well known to be highly variable among organisms, especially in bacteria covering, indeed, a very broad range from ~15 to ~75 % of GC, i.e. the molar ratio of guanine and cytosine ([1] and references therein). However, high or low GC levels are not without effect on the DNA molecule. Bio-physic studies carried out on the DNA structure showed that high GC content levels confer to the molecule an increased flexibility, or bendability [2]. Using a different approach, the result was recently confirmed by Babbitt and Schulze [3].

The effect of the GC content on the DNA structure opened new perspectives regarding the nature of the forces driving the base composition variability among and within genomes. In fact, GC-richer DNA on one hand can better tolerate the torsion stress (produced for example during the transcription process), on the other shows lower propensity to the nucleosome formation potential (NFP) than the AT-rich ones [4, 5]. Hence, the DNA would be more prone to have an open configuration structure, easily accessible to the transcriptional complex [6]. Both bendability and NFP were the pillars on which the metabolic rate hypothesis was grounded [6], further supported by an increased expression level from GC-poor to GC-rich genes [7, 8].

Testing the above hypothesis on teleostean fishes, the routine metabolic rate (temperature-corrected by Boltzmann’s factor according to Gillooly and colleagues [9]) turned out to be significantly correlated with the average genomic GC content, both decreasing from polar to tropical habitat [10], a decreasing not dictated by a dissimilar rate of the methylation-deamination process of the CpG doublets [11]. However, the above results were not taking into account the effect of the environmental salinity and nor the level of activity in relation with specific lifestyle, such as migration, on the routine metabolic rate of each species. Teleosts, indeed, are widely distributed in all aquatic environments: freshwater species (FW) populate all the inland waters, from river to lakes and ponds, while seawater species (SW) populate oceans and seas. The osmotic concentration is well known to be very different between the two environments ranging, indeed, from 1 to 25 mOsmol · kg^−1^ in freshwater, and being ~1000 mOsmol · kg^−1^ in seawater [12]. In spite of that, all teleosts share almost the same internal fluid concentration, ranging from ~230 to ~300 mOsmol · kg^−1^ [12]. Consequently, the osmotic deltas between internal and external medium in FW and SW are different, being  ~300 and  ~700mOsmol · kg^−1^, respectively [12].

The pioneering methods developed in order to quantify the amount of energy required in the osmoregulatory process were grounded on the following intuitive model: a lower osmotic delta (between internal and external fluids) should have been less energetically demanding.

Along this line, acclimative studies were performed with the aim to clarify if the hypo-osmoregulation of SW was more costly than the hyper-osmoregulation of FW [13]. Unfortunately, no clear cut conclusions were reached, and the following criticisms were raised against the acclimative approach: i) only a small number of species are capable to adapt to large salinity ranges [14]; and ii) the acclimation to different salinity involves other energy-consuming processes not directly coupled with the osmoregulation *per se*, such as the hormonal cascade produced by the osmosensing and acclimation processes [15].

A different approach to the problem of the energetic cost of the osmoregulatory process was developed by Kirschner [16, 17]. Indeed, taking advantage from previous measurements of ions concentrations in the organs individuated as the regulatory ones (i.e. gills and gut), knowing the principal mechanism of passive and active ion movements, and calculating the theoretical number of the ATP molecules spent to maintain the different osmolarities between internal and external fluids, Kirschner reached the conclusion that the hypo-osmoregulatory process was more energetically demanding than the hyper-osmoregulatory one [16, 17]. However, also the Kirschner’s approach was not criticisms less, since the energetic cost of the osmoregulatory process measured on isolated organs could lead to different conclusions compared to the measurement performed using the whole living animal [18].

Independently from the above line of research, several studies on teleost fishes highlighted that a very active lifestyle (such as that of migratory and/or pelagic species) would affect the metabolic rate and some morphological traits, such as the gill area.

Hughes in his pioneering studies, indeed, first provided evidence showing that “more active” species tend to have larger gill surface and shorter diffusion distances than " less active" ones ([19, 20], for a review of Hughes’ works). The topic of gill feature was further analyzed by De Jager and Dekkers [21], showing that gill area and oxygen uptake were positively correlated. Moreover, the same authors observed that the more active SW species also showed higher oxygen uptake, a link barely discernible in FW ones [21]. In subsequent analyses carried out on a few species, SW were reported to be characterized by more extended gill area than FW [22]. Moreover, the same authors proposed that the more active species among SW should have extended gill area and higher metabolic rate [22]. Recently, Friedman and coworkers [23] reported that the adaptation of demersal fish species to the Oxygen Minimum Zone in Monterey Canyon (California) is determined by increased gill surface area rather than enzyme activity levels.

On the other hand, osmoregulation poses a constraint on gill area, as an increase of this area would increase diffusional ion uptake, for SW species, or loss, for FW ones [24]. This would carry a constraint on the activity-metabolic rate relationship, which will be more dependent on environmental salinity.

The above results prompt us to reanalyze the oxygen consumption of teleostean fishes, taking into account two factors mainly affecting the physiology and the morphology of teleosts: the environmental salinity and the different migratory skills. Hence, the aim of the present work is to test if both salinity and migration significantly affect the routine metabolic rate and average genomic GC content. Indeed, as stressed above, the correlation between the two variables represents the keystone of the metabolic rate hypothesis [4, 6]. Moreover, since the gill area has been reported to be a major morphological expression of metabolic rate [19], this parameter was also analyzed according to the two kind of lifestyles. The results clearly highlight that active species living in seawater show coincidentally the highest routine metabolic rate, the highest specific gill area, and the highest average genomic GC content. Thus, opening to an interesting liaison among basic genome feature (i.e. the GC content), physiology (i.e. the routine metabolic rate), and morphology (i.e. gill area) of teleosts.

## Methods

Reports regarding the salinity of the habitat, the migratory performances and the gill area of teleostean fishes were retrieved from www.fishbase.org [25]. Species with conflicting information about salinity and/or migration were discarded, namely: *Aphanius dispar dispar, Aphanius fasciatus, Ciprinodon variegatus, Fundulus heteroclitus, Lagodon rhomboides, Leptococcus armatus, Takifugu rubripes*, *Bathygobius soporator* and *Perca fluviatilis*. Species with no indications about migration were considered non-migratory.

Values of the routine metabolic rate were retrieved from literature [10], whereas those regarding *Corydoras aeneus* and *Tetraodon nigroviridis* were determined according to the procedures described in [26]. For each species the routine mass specific metabolic rate values, expressed as milligrams of oxygen consumed per kilogram of wet weight per hour (mgxkg^−1^xh^−1^), were temperature-corrected using the Boltzmann's factor MR = MR_0_e^E/kT^, where MR is the temperature-corrected mass specific metabolism, MR_0_ is the metabolism at the temperature T expressed in °K; E is the energy activation of metabolic processes ∼ 0.65 eV; k is the Boltzmann’s constant =8.62 Å ~ 10^−5^ eV K^−1^ [9]. The MR values were ln-normalized. The final dataset consisted of 196 species belonging to 75 teleostean families (Additional file 1: Table S1).

Regarding the specific gill area (Gill), the value of each species was expressed as cm^2^xg^−1^, i.e. the ratio between the gill area and fresh body mass. If more than one value was available for a given species the median was used. The final dataset comprises 108 species, covering 56 families of teleostean fishes (Additional file 2: Table S2).

Regarding the GC content, data were retrieved from current literature [27–30]; see supplementary materials for detailed information. The final dataset consisted of 227 species covering 69 families of teleostean fishes (Additional file 3: Table S3).

Gene expression data of green spotted pufferfish *Tetraodon nigroviridis* [31] were downloaded from ArrayExpress [32]. The corresponding gene coding sequences were retrieved from the Genoscope site (http://www.genoscope.cns.fr). Length and base composition were calculated for each sequence and merged with the log-transformed expression data. Sequences containing unknown nucleotides or shorter than 100bp were removed. Moreover, considering the GC variability along genes [33], CDSs lacking of ATG start codon and/or the ending stop codons were discarded. The final dataset accounted for 8317 unique CDSs. Under the implicit assumption that a correlation holds between the GC levels of CDSs and isochores, the CDS dataset was split in four groups according to the GC content of the isochores.

### Statistical analyses

Mann–Whitney and two-way ANOVA tests were used to assess the statistical significance of the differences. Regarding the two-way ANOVA, the significance of the main effects and the interaction effect was assessed non parametrically by bootstrap (10^3^ resampling), thus relaxing the assumption of normality. The statistical analysis was implemented in R and it is provided as supplementary material in the R-markdown form in the spirit of reproducible research [35]. The significance of different expression levels observed within the green spotted pufferfish genome was assessed by the Kruskal-Wallis test.

## Results

**Fig. 1:**
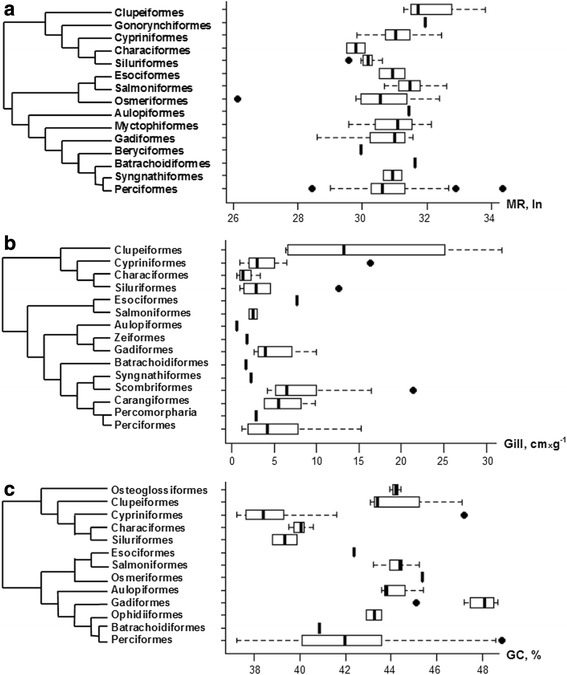
Cladograms based on the phylogenetic tree reconstruction by [37] showing the relations among the orders comprised in this study. The boxplot shown the distribution of the specific values within each order for the routine metabolic rate (panel **a**), specific gill area (panel **b**), and genomic GC content (panel **c**)

Clarke and Johnston [36] observed no effect of phylogeny on the routine metabolic rate of teleosts. However, their conclusion was biased by the absence of a robust phylogenetic tree. Hence, we tackled the topic using a very recent tree reconstruction of teleostean species [37]. According to Clarke and Johnston [36], in order to have a reliable number of observations along the tree branches, species were grouped at order level. Values of routine metabolic rate temperature and mass-corrected (MR), gill area (Gill) and average genomic GC-content (GC%) were calculated for each order present in our databases and showed as box plot (Fig. 1). Present results confirmed the observation of Clarke and Johnston [36], since no phylogenetic signal was observed for the routine metabolic rate (Fig.1, panel a). Indeed, the variation of MR within the order of Perciformes was covering quite the entire range or variability shown by the all teleostean species. Considering Gill, although if a great variability was observed among orders, no significant differences were found in pairwise comparisons according to the Mann–Whitney test Bonferroni - corrected for multiple tests. Hence, also in the case of Gill no phylogenetic signal was observed (Fig.1, panel b). The same conclusion also applied for the GC% (Fig.1, panel c), in very good agreement with previous reports by Bernardi and Bernardi [38].

**Fig. 2:**
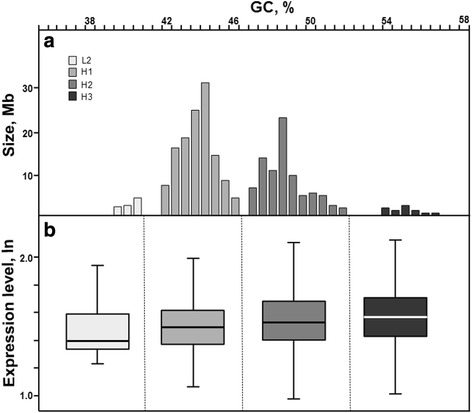
Genome organization of *Tetraodon nigroviridis* (modified from [34]) (panel **a**). Boxplot of the gene expression level (*p*-value < 4.1 × 10^−15^ by Kruskal-Wallis test) (panel **b**). Dotted lines represent the limits used to split the expression level database

To this regards, it is worth to bring to mind that teleosts are characterized by a peculiar compositional evolution mode. Indeed, differently from high vertebrates, where increments of the GC%, as for example from amphibians to mammals [39, 40], are paralleled by increments of the within genome base composition variability (also known as the transition mode of evolution), in fishes increments of GC% from one species to another are paralleled by a whole-genome shift (also known as the shifting mode of evolution) [41, 42]. In spite of a marked homogeneity of fish genomes, characterized by the main presence of two isochores [34], bendability and nucleosome formation potential were both shown to significantly correlates with the GC content of exons, introns and 10 kb of DNA stretches [4, 43]. Here we checked the expression level analyzing data available for *Tetraodon nigroviridis*. The results reported in Fig. 2, clearly showed a significant different average gene expression level among the four isochores described in the green spotted pufferfish genome (*p*-value < 4.1 × 10^−15^ by the Kruskal-Wallis test). Significant differences were also found restricting the analysis between the two main isochores H1 and H2 (*p*-value < 6.8 × 10^−5^ by the Mann-Whitney test).

**Fig. 3:**
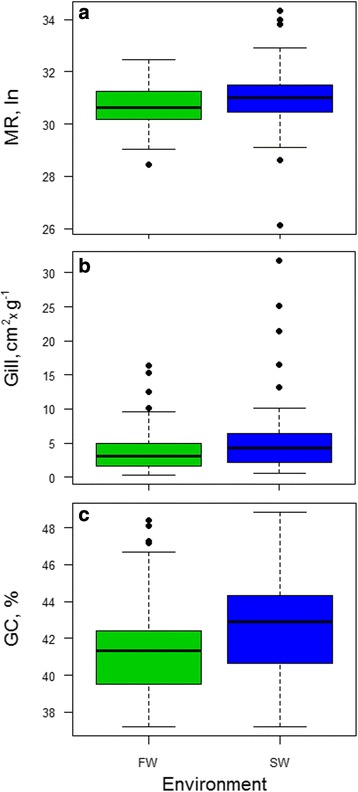
Boxplot of routine metabolic rate (panel **a**), specific gill area (panel **b**), and genomic GC content (panel **c**) for freshwater (FW) and seawater (SW) species

Classical multivariate statistics, such as the Principal Components Analysis, could not be used for the study of the three variables: MR, Gill and GC%. Indeed, the intersection of the three datasets accounted for only 12 species. Therefore, on the basis of the environmental salinity, each independent dataset was first split in two major groups: i) FW, grouping teleosts spending the lifecycle mainly in streams or ponds (i.e. all the species whose range of habitats is freshwater or freshwater-brackish, and the catadromous species); and ii) SW, grouping teleosts spending the lifecycle mainly in oceans (i.e. marine, marine-brackish plus the anadromous species). The specific routine metabolic rate, temperature-corrected using the Boltzmann’s factor (MR), the specific gill area expressed in cm^2^xg^−1^ of body mass (Gill), and the average genome base composition, i.e. GC content (GC%), were computed and compared between FW and SW by the Mann–Whitney test. All pairwise comparisons showed the same trend. Indeed, MR, Gill and GC% were higher in SW species (Fig. 3). The *p*-values of each FW *vs* SW comparison were <1.0 × 10^−2^, <5.7 × 10^−2^ and <1.8 × 10^−4^, respectively.

**Fig. 4:**
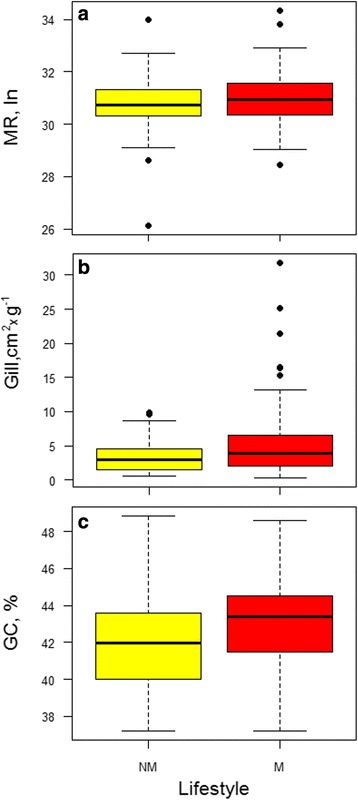
Boxplot of routine metabolic rate (panel **a**), specific gill area (panel **b**), and genomic GC content (panel **c**) for non-migratory (NM) and migratory (M) species

In order to assess if a different lifestyle could also affect MR, Gill and GC%, the three independent datasets were split in two categories: migratory species (M), grouping catadromous, potamodromous, amphidromous, oceanodromous and anadromous, and non-migratory species (NM). The former showed higher MR, Gill and the GC% then the latter (Fig. 4, panels a, b and c). The corresponding *p*-values, according to the Mann–Whitney test, were <7.9 × 10^−2^, <3.8 × 10^−2^ and <6 × 10^−3^, respectively. In literature a significant positive correlation was reported to hold between the routine metabolic rate and the maximum metabolic rate [44, 45]. In other words, species with a larger capacity for highly costly activities, including migration, would have not only a high routine metabolic rate [44, 45], but also an extended gill area [21, 22]. On the basis of this expectation, the one-tail Mann–Whitney test was applied in the comparison of migratory and non-migratory species regarding both MR and Gill variables.

**Fig. 5:**
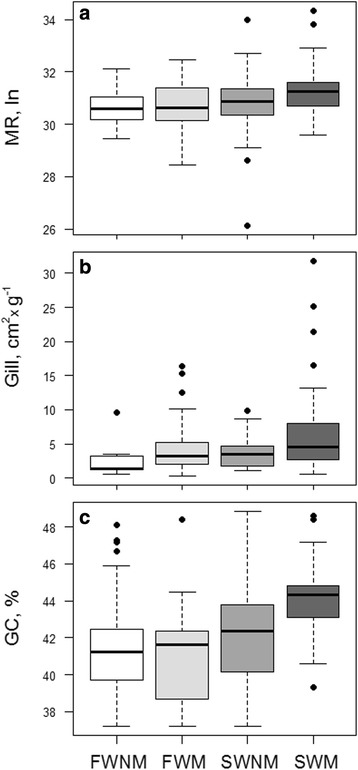
Boxplot of routine metabolic rate (panel **a**), specific gill area (panel **b**), and genomic GC content (panel **c**) for freshwater non-migratory (FWNM), freshwater migratory (FWM), seawater non-migratory (SWNM) and seawater migratory (SWM) species

The combined role of salinity and migration on the three measured variables, was assessed by partitioning each data set in four sub-groups: both freshwater and seawater species were split in non-migratory and migratory categories, namely FWNM, FWM, SWNM and SWM. The corresponding box plots were reported in Fig. 5 (panels a, b and c, respectively). In each panel, the medians of the four subgroups showed the same trend, specifically increasing from FWNM to SWM (Fig. 5; see also Table 1). Unfortunately, within each dataset the four categories were not equally represented, and a normal distribution was not found (Shapiro-Wilk normality test *p*-value <5 × 10^−5^). Thus, to assess the significance of the differences, if any, a two-way ANOVA test with bootstrap was performed. The *p*-value was calculated as ∑I (Resampling F-values > Real F-value)/1000, where I() denotes the indicator function.

**Table 1:** Medians for each group

	Gill, cm^2^xg^-1^	MR, ln	GC, %
FWNM	1.41	30.58	41.22
FWM	3.24	30.63	41.62
SWNM	3.44	30.85	42.37
SWM	4.61	31.26	44.31

The results (Fig. 5; panels a, b and c) showed that among the four groups:

i) migration was significantly affecting all the three variables. The *p*-values, indeed, were <4 × 10^−3^ for the MR, <7 × 10^−3^ for the Gill, and <6 × 10^−3^ for the GC%;
ii) environmental salinity was affecting MR and GC%, but not Gill (*p-*value <2.5 × 10^−2^, <1 × 10^−6^ and <12.2 × 10^−1^, respectively);
iii) the combined effect of salinity and migration was affecting mainly the GC% (*p*-value < 2.9 × 10^−2^), slightly the MR (*p*-value < 8.1 × 10^−2^), and not at all the Gill (*p*-value < 80 × 10^−1^).

Very interestingly, the SWM group of fishes, the ones characterized by the most energetically expensive lifestyle, showed coincidentally the highest MR, the highest Gill and the highest GC% (Fig. 5; panels a, b and c, respectively). According to the multiple hypothesis test [46], the converging effect of salinity and migration on the three variables was statistically significant, *p*-value <3.1 × 10^−2^.

## Discussion

Does the routine metabolic rate is higher in seawater than freshwater fishes? This question, that has been matter of a long debate grounded on many different experimental and theoretical approaches [18] for a review, find a positive answer in the present study. The consistency of this result (*p*-value <1.0 × 10^−2^) rely on the analysis of ~200 species of teleosts. Such a huge comparison (based on species characterized by different body mass and living in habitats with different environmental temperature) have been possible due to the normalization of the data about the routine metabolic rate by the Boltzmann’s factor, according to the equation MR = MR_0_e^E/kT^ [9]. The result was further supported by the analysis of the phenotypic character mainly linked to the metabolic rate, namely the specific gill area [19]. Indeed, analyzing an independent dataset of >100 teleosts, SW species turned out to have a specific gill area higher than those of FW ones (*p*-value <5.7 × 10^−2^). Hence, there was a very good accordance between morphology and physiology in favor of the SW species. In the light of the metabolic rate hypothesis [4, 6], species showing a high metabolic rate should also show a high GC content. Thus the expectation would have been that the average genomic GC content of SW species would be higher than FW ones. In teleosts, the inter-genomic correlation between the two variables was found to significantly hold [10]. The link between metabolic rate and GC content obviously is not straightforward, but goes through a consideration about the DNA structure. Indeed, to be GC- or AT-rich is not equivalent for the DNA molecule [5]. Structural analyses performed independently with two different approaches reached, in fact, the same conclusion: a GC-richer DNA is more suitable to cope the torsion stress [2, 3]. Duplication and transcription are the two main functional steps during which the DNA molecule is under torsional stress because the opening of the double helix. Noticeably, the duplication process cannot be invoked, since it is well known that a great GC content variability have been observed not only among organisms ([1, and references therein), but also within genomes, well known to be a mosaic of genome regions with different GC content, i.e. isochores [40, 41]. Thus, the transcription process should be considered as the main factor of the torsion stress affecting the DNA structure [4]. Several studies, indeed, support the correlation between GC content and the transcription levels. In fact, the *in situ* hybridization of GC-rich and GC-poor probes showed that human GC-rich regions, harboring GC-rich genes, were in an open chromatin structure [47]. Besides, human GC-rich genes showed transcriptional levels significantly higher than those of GC-poor ones [8]. Moreover, according to the KOG classification of genes [48], several mammalian genomes were analyzed showing that genes involved in metabolic processes were, at the third codon positions, GC-richer than those involved in information storage or in cellular processes and signaling [49]. Present results highlighted that also within the genome of green spotted pufferfish GC-rich genes showed higher transcriptional levels than GC-poor ones.

In the line of the above considerations and results, and keeping in mind that a significant correlation between MR and GC content was already observed among teleosts [10, 11], was not a mindless expectation that the GC content of SW would have been significantly higher than that of FW, and, indeed, the *p*-value was <1.8 × 10^−4^. Although the difference seems to be in a very little order of magnitude, hence apparently negligible from an evolutionary point of view, detailed analysis on five teleostean species (i.e. zebrafish, medaka, stickleback, takifugu and pufferfish) showed that small differences of the average genome base composition hide great differences at the genome organization level. Indeed, comparing the genomes of stickleback and pufferfish (showing an average genomic GC content of 44.5 and 45.6 %, respectively), the genome of the latter was characterized by the presence of very GC-rich regions (isochores) completely absent in the former [34]. It is worth to recall here that in teleosts the routine metabolic rate, not only was found to correlate significantly with the genomic GC content, as mentioned above [10], but also to affect the genome features. Indeed, analyzing five full sequenced fish genomes, increments of MR were found to significantly correlate with the decrease of the intron length [50].

The comparison of migratory (i.e. catadromous, potamodromous, amphidromous, oceanodromous and anadromous) and non-migratory species showed that the specific gill area of migratory species was significantly higher than that of non-migratory ones (*p*-value <3.8 × 10^−2^) and the GC% showed the same statistically significant trend (*p*-value <6 × 10^−3^), being higher in the migratory group. However, the difference of MR, higher in the migratory group, was at the limit of the statistical significance (*p*-value <7.9 × 10^−2^). Thus, in order to disentangle the effect of the environmental salinity from that of the migratory attitude, the three datasets concerning MR, Gill and GC% were split in four groups, namely freshwater non - migratory (FWNM), freshwater migratory (FWM), seawater non - migratory (SWNM) and seawater migratory (SWM). At first glance, among the four groups a good agreement was observed regarding the three variables, showing, indeed, increasing average values from FWNM to SWM (Fig. 5). However, the two-way ANOVA test showed that the variation among the four groups was significantly affected by both the environmental salinity and the migratory attitude only regarding MR and GC content (Fig. 5; panels a and c), while Gill was significantly affected only by migration and not by the environmental salinity (Fig. 5: panel b). The combined effect of a costly osmoregulation and the need for a high scope for aerobic metabolism would justify the higher MR in marine migratory fish species. Moreover, the need of an adequate oxygen uptake in active species (such as migratory species) is a major determinant of gill area. It is worth to note that an increase in gill area is disadvantageous for osmoregulation, particularly for freshwater species, as it increases the obligatory ion exchanges and the energetic cost of compensating them [24, 51]. This would explain the observed discrepancy between MR and Gill dependency from migratory habit and salinity. Nevertheless, the multiple hypothesis test [46] showed that the SWM group was significantly the highest for all the three variables. Therefore, in the teleost group, that is under the highest environmental demanding conditions due to both salinity and migration, the three variables converged reaching the highest values. On one hand, present results supported previous reports on both metabolic rate and gill area [21, 22], on the other opened to new genomic perspective since, as far as we know, this is the first report that phenotypic, physiological and genomic feature are linked under a common selective pressure. Interestingly, the genomic feature, i.e. the average GC content, was a very “reactive” variable to environmental changes. Indeed, according the two-way ANOVA test, the GC% was the only variable being simultaneously affected, and by environmental salinity and migration attitude, *p*-value <2.9 × 10^−2^. Such “reactivity” was not observed for both Gill and MR, most probably because morphological/functional and physiological constraints.

## Conclusion

Certainly the present analyses of metabolic rate, gill area and genomic GC-content carried out on teleostean species could not be considered as a demonstration of the cause-effect link between metabolism and DNA base composition. Nevertheles, represents a further support to the metabolic rate hypothesis proposed by Vinogradov [5, 6], underlining that the torsion stress, proposed to be the factor responsible of the GC increment, could be not such a mysterious selective force.

## Ethics approval and consent to participate

In vivo experiments were performed at the Biology Department of the University of Naples Federico II, following the procedures approved by the Institutional Animal Care and Use Committee (IACUC) of the University of Naples Federico II, Naples, Italy.

## Consent for publication

Not applicable.

## Availability of data and material

The datasets supporting the conclusions of this article are included within the article and its additional files.

## Additional files

Additional file 1: Table S1. Basal metabolic rate body mass- and temperature-corrected by Boltzman’s factor. (XLSX 25 kb) Additional file 2: Table S2. Gill area. (XLSX 70 kb) Additional file 3: Table S3. Genomic GC value (%), environmental and lifestyle data for the species used in the analyses. (XLSX 19 kb)

## Abbreviations

GC%: Molar ratio of Guanine and Cytosine
NFP: Nucleosome formation potential
FW: Freswater teleosts, i.e. all species whose range of habitats is freshwater or freshwater-brackish, and the catadromous species
SW: Seawater teleosts, i.e. marine, marine-brackish plus the anadromous species
MR: Natural log mass specific routine metabolic rate/temperature-corrected using the Boltzmann’s factor
Gill: Ratio between gill area and fresh body mass
M: Migratory teleosts, i.e. catadromous, potamodromous, amphidromous, oceanodromous and anadromous
NM: Non-Migratory teleosts
FWNM: Freshwater non-migratory teleosts
FWM: Freshwater migratory teleosts
SWNM: Seawater non-migratory teleosts
SWM: Seawater migratory teleosts
